# Comparative Transcriptome Analysis During the Seven Developmental Stages of Channel Catfish (*Ictalurus punctatus*) and Tra Catfish (*Pangasianodon hypophthalmus*) Provides Novel Insights for Terrestrial Adaptation

**DOI:** 10.3389/fgene.2020.608325

**Published:** 2021-01-21

**Authors:** Xiaoli Ma, Mei Shang, Baofeng Su, Anne Wiley, Max Bangs, Veronica Alston, Rhoda Mae Simora, Mai Thi Nguyen, Nathan J. C. Backenstose, Anthony G. Moss, Thuy-Yen Duong, Xu Wang, Rex A. Dunham

**Affiliations:** ^1^School of Fisheries, Aquaculture and Aquatic Sciences, Auburn University, Auburn, AL, United States; ^2^Alabama Agricultural Experiment Station, Auburn, AL, United States; ^3^Department of Anatomy, Physiology and Pharmacology, Auburn University, Auburn, AL, United States; ^4^Department of Biological Science, Florida State University, Tallahassee, FL, United States; ^5^College of Fisheries and Ocean Sciences, University of the Philippines Visayas, Miagao, Philippines; ^6^College of Aquaculture and Fisheries, Can Tho University, Can Tho, Vietnam; ^7^Department of Biological Sciences, University at Buffalo, Buffalo, NY, United States; ^8^Department of Biological Sciences, Auburn University, Auburn, AL, United States; ^9^Department of Pathobiology, Auburn University, Auburn, AL, United States; ^10^HudsonAlpha Institute for Biotechnology, Huntsville, AL, United States

**Keywords:** air-breathing, mRNA-seq, tra catfish, terrestrial adaptation, low-oxygen tolerance

## Abstract

Tra catfish (*Pangasianodon hypophthalmus*), also known as striped catfish, is a facultative air-breather that uses its swim bladder as an air-breathing organ (ABO). A related species in the same order (Siluriformes), channel catfish (*Ictalurus punctatus*), does not possess an ABO and thus cannot breathe in the air. Tra and channel catfish serve as great comparative models for investigating possible genetic underpinnings of aquatic to land transitions, as well as for understanding genes that are crucial for the development of the swim bladder and the function of air-breathing in tra catfish. In this study, hypoxia challenge and microtomy experiments collectively revealed critical time points for the development of the air-breathing function and swim bladder in tra catfish. Seven developmental stages in tra catfish were selected for RNA-seq analysis based on their transition to a stage that could live at 0 ppm oxygen. More than 587 million sequencing clean reads were generated, and a total of 21,448 unique genes were detected. A comparative genomic analysis between channel catfish and tra catfish revealed 76 genes that were present in tra catfish, but absent from channel catfish. In order to further narrow down the list of these candidate genes, gene expression analysis was performed for these tra catfish-specific genes. Fourteen genes were inferred to be important for air-breathing. Of these, *HRG*, *GRP*, and *CX3CL1* were identified to be the most likely genes related to air-breathing ability in tra catfish. This study provides a foundational data resource for functional genomic studies in air-breathing function in tra catfish and sheds light on the adaptation of aquatic organisms to the terrestrial environment.

## Introduction

Oxygen is indispensable for all aerobic creatures. For animals, breathing is a critical biophysical and voluntary process that involves the uptake of oxygen and transferring it to the cells. The transport and consumption of oxygen involves multiple physiological and biochemical processes. For fish that receive oxygen from their surrounding aquatic environment, gills serve as the primary site for gas exchange (Palzenberger and Pohla, 1992). In addition to their respiratory function, gills also serve as a pathway for the exchange of non-volatile molecules between the blood and the environment, and most fish exchange gases through gills that are protected under an operculum on both sides of the pharynx (Olson, 1991). In addition to gill-based breathing, there are many fish species that can perform aerial breathing (Barrell, 1916). Air-breathing fish are fish that can undergo gas exchange directly with the atmosphere, rather than in water. Primitive fish were the first vertebrates to breathe the atmosphere air, in addition to aquatic respiration (Hedrick and Katz, 2015). Air-breathing fish utilize many unique behaviors to access atmospheric oxygen, including rising to the surface of water and gulping air. Some of them even crawl onto land where they are able to survive for a long time (Graham, 1997). Although there are a number of different morphological adaptations for air-breathing, all display a thin barrier between gas and blood, usually in the form of a large pleated membrane (Maina, 2002). The membrane creates small diffusion distances for gases and decreases branchial vascular resistance and also increases the respiratory surface area, resulting in high diffusion capacity in air-breathing fish and improved respiratory efficiency, allowing such fish to survive even being exposed to air (Maina, 2002).

There are more than 370 extant air-breathing fish species in 49 families. The air-breathing organs (ABOs) among these fish vary considerably (Graham and Lee, 2004). For species that use modified gills as an ABO, such as *Clarias macrocephalus* and *C. batrachus*, the efferent branchial arteries of the anterior (first and second) gill arches serve as the accessory ABO and are also the site for gas exchange. The ventral aorta exits the heart and splits into a ventral and dorsal branch. The ventral branch supplies blood to the anterior gill arches, which then flows through the accessory ABO and back to the heart. The dorsal branch distributes blood to the posterior (third and fourth) gill arches and proceeds to the circulatory system, which transports oxygen-rich blood to other tissues (Olson et al., 1986). In some species, such as *Misgurnus anguillicaudatus* and *Corydoras aeneus*, a modified intestine serves as the ABO. In these species, air is taken into the mouth with unidirectional ventilation of the posterior region of the intestine and continuous exhaust of gas from the vent (Mcmahon and Burggren, 1987). In the posterior region of the intestine, the mucosa has very smooth surfaces and is lined with respiratory epithelium and capillary networks, and a thin air–blood barrier (0.24–3.00 μm) is the site of air exchange (Podkowa and Goniakowska-Witali?ska, 2002). Intestinal gas exchange and digestion in fish are not mutually exclusive processes. Animals can feed and breathe air simultaneously. In *M. anguillicaudatus*, digestion occurs in the anterior region of the gut, while air-breathing occurs in the posterior region; fecal matter is controlled by wrapping it in a mucous sheath (Mcmahon and Burggren, 1987).

The modified swim bladder is another ABO. The teleost swim bladder is a large, well-vascularized organ and is widely considered as evolutionarily homologous to the lungs (Alexander, 1966; Nguyen, 2015). The swim bladder can serve as a gas container and can function as a hydrostatic organ, allowing the fish to maintain itself in a vertical position in the water column. The swim bladder also serves as a resonating chamber to produce or receive sound. Orders of fish using the swim bladder for aerial respiration include the *Gonorynchiformes* (Motta, 1984), *Characiformes*, and some species in Siluriformes, such as the *Pangasianodon hypophthalmus* (tra catfish; Graham et al., 1978; Liem et al., 1984). Tra catfish are facultative air-breathers. Their swim bladder extends from the posterior of the head to the tail beyond the anus. Collagen-rich fibrous walls form subdivisions, supporting the swim bladder. There are two types of epithelial cells on the surface of the fibrous wall. One is a thin respiration epithelium, which covers the majority of the surface and is highly vascularized, which serves as a major gas exchange region between air and blood in the swim bladder. The second cell type is thicker with a brush border, which also supports the structure of the swim bladder (Phuong et al., 2018). Phuong et al. found that the volume and the respiratory surface area of the swim bladder in tra catfish are strongly and positively correlated with body mass.

Channel catfish (*Ictalurus punctatus*), which can only obtain oxygen from water with gills, and its hybrid from mating with the blue catfish (*I. furcatus*) are recognized as the most extensively cultured type of catfish in the United States. The culture of tra catfish (*P. hypophthalmus*), a facultative air-breather, accounts for two-thirds of Vietnam’s overall aquaculture yields (Nguyen et al., 2014).

The Mekong Delta in Vietnam has become one of the world’s largest aquaculture regions, with an annual output of tra catfish reaching 1.14 million tons and an export income of approximately 1.4 billion dollars (De Silva and Phuong, 2011). The air-breathing capability of tra catfish allows them to live under hypoxic conditions. As a result, tra catfish have a substantial advantage over channel catfish, which cannot breathe air and require high-oxygen, aerobic environments (Burleson and Smatresk, 2000; Lefevre et al., 2011).

In practical catfish production, hypoxia is a frequent and significant problem, resulting in enormous economic losses. Aerators are extensively used in the United States catfish farming industry, but are subject to mechanical failure or human error and are expensive, energy-intensive operations. A better understanding of the mechanisms for tolerating hypoxia is critical for continued successful United States catfish aquaculture productivity, especially facing competition from Southeast Asia and as climate change forces United States production to deal more actively with temperature-induced hypoxia. In addition, the study of hypoxia tolerance and the physiological and structural changes and molecular basis for understanding how this has occurred will better guide us for exploring the evolution of life from the ocean to the terrestrial environment.

In this study, hypoxia challenge and histology experiments were conducted to reveal the development of the swim bladder in tra catfish and to understand its air-breathing function better. RNA-seq analysis of seven critical stages during early development of the tra catfish associated with different levels of air-breathing ability was conducted to identify the genes and pathways leading to the development of swim bladder and functioning of air-breathing in tra catfish larvae.

## Materials and Methods

### Ethics Statement

All experimental procedures involving animal care and tissue collection were approved by the Auburn University and Can Tho University Institutional Animal Care and Use Committee (AU-IACUC, PRN#: 2016-2901 on June 13, 2016, and the Can Tho University Animal Welfare Committee on November 15, 2016). All animal-related procedures were performed following the Guide for the Care and Use of Laboratory Animals and the Animal Welfare Act in the United States and in Vietnam.

### Experimental Animals and Tissue Collection

Tra catfish embryos were produced at Can Tho University, Vietnam. Tra catfish embryos were hatched within 1 day post fertilization, and samples were collected every 24 h over a 30-day period after hatching. A total of 20–50 eggs/embryo/fry were collected as two sets at each sampling and later used for histological and gene expression analysis (Backenstose, 2018). At each sampling point, one set of fry was euthanized with 200 ppm buffered MS-222 and stored in RNAlater solution (Thermo Fisher Scientific). Samples were shipped to the United States on dry ice and stored at −80°C until RNA isolation was carried out. The second set of fry was sampled in the same way, placed in 10% neutral buffered formalin, and sealed with screw top lids (Backenstose, 2018). All samples for histology were maintained at room temperature until use.

### Low-Oxygen (Anoxia) Challenge

Anoxia, 0 ppm dissolved oxygen (DO), challenge was conducted at Can Tho University, Vietnam, to determine the tolerance of tra catfish to anoxia conditions at a temperature of 27°C. Tra catfish larvae were challenged each day from 2 to 12 days post fertilization. One group of 20 larvae was placed in a 2-L container with oxygen supply as a control treatment. The other group was stocked in a second 2-L container at first with aeration, and then the DO level was lowered by bubbling nitrogen gas into the water until a 0-mg/L DO concentration was obtained as measured by a DO meter. DO levels, larval behavior, and survival rate were measured and recorded every 15 min. The experiment was repeated daily until all 20 fish survived in the oxygen-eliminated container (the fish demonstrated facultative air-breathing) after 12 days post fertilization.

### Histological Analysis

Tra catfish samples for each life stage were removed from 10% formalin and used for paraffin processing for embedment and subsequent sectioning. Following dehydration by graded ethanol, hyalinization, and infiltration by dimethylbenzene with a published protocol (Fischer et al., 2008), samples were embedded in paraffin, and subsequently 7-μm-thick transverse sections were made. Sections went through a water bath at 40°C and then placed on glass slides. The slides were kept in an incubator at 37°C overnight. Following deparaffination by xylene substitution in HEMO-DE, slides were then stained with hematoxylin and eosin (H&E) according to standard procedures. Following H&E staining, slides were covered with a cover slip and left for 48 h at room temperature to dry. Slides were observed and imaged with an Olympus^®^ BHS fluorescence binocular microscope equipped with a 3.4-megapixel color digital camera (Qimaging^®^ model Micropublisher 3.3 RTV). Image-Pro Plus 7 software (Media Cybernetics, Bethesda, MD, United States) was used to capture the image.

### RNA Isolation, Library Construction, and Sequencing

Samples for transcriptomic analysis were taken at 2, 4, 6, 8, 9, 10, and 11 days post fertilization (dpf) based on the results of the low-oxygen challenge experiments. At each time point, two replicates (pooled from four embryos/larvae) were taken for RNA isolation and Illumina sequencing. Sample collection, processing, and total RNA extraction were described in our previous study (Ma et al., 2020). RNA concentration and integrity of each sample were initially measured on a NanoDrop 2000 Spectrophotometer (NanoDrop Technologies). For each life stage, equal amounts of RNA from the two pooled replicates was used for RNA-seq library construction and RNA sequencing.

Library preparations and sequencing reactions were conducted at GENEWIZ, LLC (South Plainfield, NJ, United States). Ribosomal RNA was depleted *via* the Ribozero rRNA Removal Kit (Illumina, San Diego, CA, United States), so that it would not interfere with the analysis of mRNA. The NEBNext Ultra RNA Library Prep Kit was utilized for Illumina RNA sequencing library preparation by following standard protocols (NEB, Ipswich, MA, United States). Amplified library yields were validated using the Agilent Tapestation 4200 (Agilent Technologies, Palo Alto, CA, United States) and quantified using Qubit 2.0 Fluorometer (Invitrogen, Carlsbad, CA, United States). The sequencing libraries were multiplexed with eight samples clustered per lane and sequenced using a 2 × 150 paired-end (PE) configuration.

### Comparative Genomic Analysis of Channel Catfish and Tra Catfish

The protein sequences of channel catfish and tra catfish were obtained from the NCBI website to determine chromosome orthology (Liu et al., 2016; Li et al., 2018; Zhou et al., 2018). The orthologs and orthogroups between channel catfish and tra catfish were identified using OrthoFinder version 2.2.7 (Emms and Kelly, 2015). In order to obtain the tra catfish-specific genes, an additional round of protein BLAST (BLASTP) was conducted for genes that are not in the ortholog groups. These genes were queried against the genes in the ortholog groups within the same species with a maximum *e*-value threshold of 1e-10. In the end, reciprocal BLASTP searches were performed to query genes with no hits from the last steps with a maximal *e*-value threshold of 1e-5. The remaining genes with no hits to any orthologs were identified as species-specific genes for subsequent analysis.

To further confirm the tra catfish-specific genes from previous steps, the species-specific genes in tra catfish were queried with the channel catfish genome using TBLASTN with a maximum *e*-value threshold of 1e-10. The recognized tra catfish-specific genes were then screened based on the percentage of identical matches (pident) and query coverage per subject (qcovs). The genes without any TBLASTN hits in the channel catfish genome were recognized as tra–catfish-specific genes present in tra catfish but absent in the channel catfish genome.

### Read Mapping and Differential Expression Analysis

Raw reads quality was evaluated in FastQC (version 0.11.5; Andrews, 2010), and low-quality bases, adapter sequences, and ambiguous nucleotides were trimmed from the raw sequences using Trimmomatic (version 0.36; Bolger et al., 2014). The reads were removed if an average sliding window Phred score over four bases was less than 25, and reads with a length shorter than 36 bases after trimming were removed. The remaining high-quality reads were used for subsequent analysis. The recently assembled tra catfish genome was used as a reference for reads mapping. The tra catfish genome was approximately 700 Mb, assembled into 568 scaffolds, with a scaffold N50 of 14.29 Mbp (Kim et al., 2018).

To profile tra catfish gene expression, tra catfish filtered reads were mapped to their genome using STAR alignment software (version 2.7.0) with a max 4% mismatching rate of the mapped length allowed and a minimum of 90% of the bases mapped to the genome (Dobin et al., 2013). HTSeq-count (Anders et al., 2015) was conducted to extract and count the read from the mapping files. After counting the number of clean reads mapped to each gene, the FPKM (fragments per kilobase of exon model per million reads mapped) method was performed for normalization, and the genes with an FPKM smaller than 0.1 were filtered out of each sample. To account for differences with the development in the two each species, differential expression analysis was performed with the R package DESeq2 (Love et al., 2014). Differentially expressed genes (DEGs) were defined as having a *P* value < 0.05 and a | log2 fold change| >1.

### Gene Ontology and Enrichment Analysis

Gene ontology (GO) terms for each differential expression comparison were obtained by using zebrafish database annotations for the unigene set as well as using clusterProfiler R software (version 3.6; Yu et al., 2012). The annotation result was then sorted with respect to biological process, cellular component, and molecular functions. clusterProfiler R was also used for a GO functional enrichment analysis of certain genes. A criterion of *P* value and *q* value cutoff of 0.05 was chosen as the threshold of significance.

### Clustering of Time Series Gene Expression Data

Clustering is universally used for gene expression data analysis. Mfuzz (Kumar and Futschik, 2007) is one of the most commonly implemented soft clustering software. A minimization of weighted square error function based on fuzzy c-means algorithm was performed to reveal structures underlying large gene expression datasets. Hard clustering approaches are preferable to identify well-separated clusters, at the cost of excluding biologically relevant genes. To solve this issue, Mfuzz soft clustering provides an overall relation between clusters, and it is more robust to noise since gene/protein clusters frequently overlap in biological data.

## Results

### Anoxia Challenge Reveals the Tolerance of Low Oxygen in Different Development Stages of Tra Catfish

**FIGURE 1:**
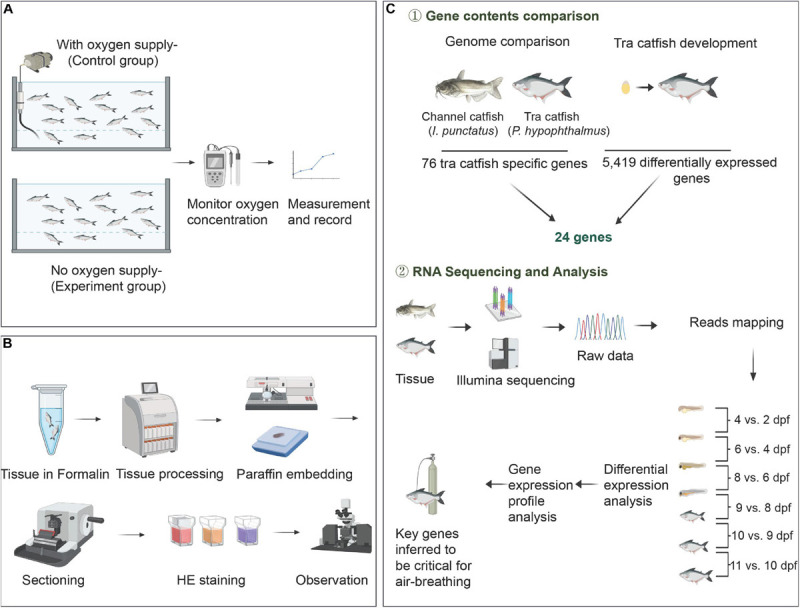
Pipeline for analysis. **(A)** Physiological analysis (hypoxia experiment) to reveal the ability of air-breathing development. **(B)** Histology experiment to illustrate the development of organs related to air-breathing. **(C)** Comparative genomic analysis and transcriptome analysis to show the key candidate genes responsible for air-breathing ability.

**FIGURE 2:**
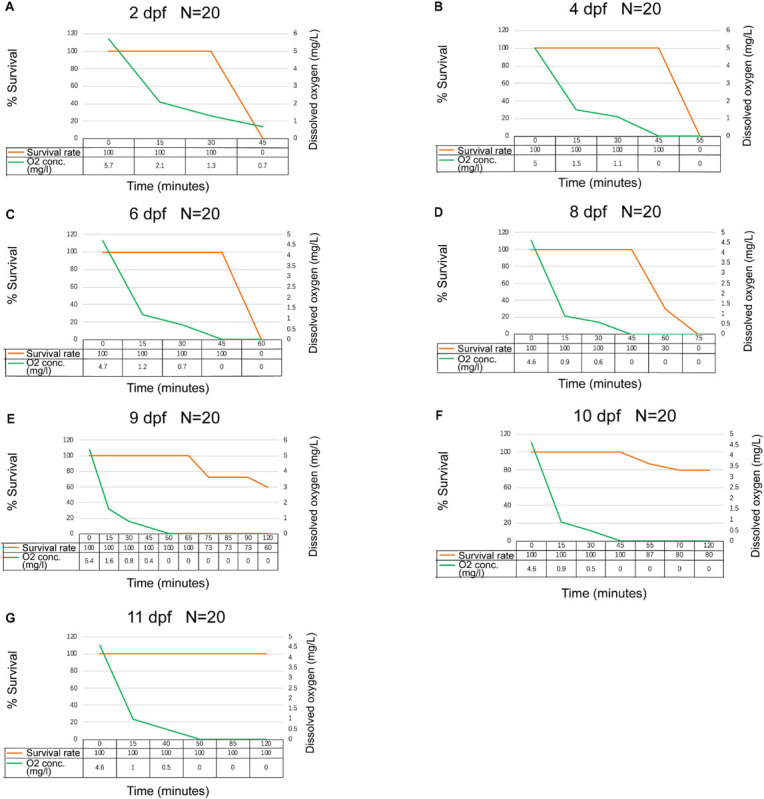
Oxygen depletion challenge for tra catfish (*Pangasianodon hypophthalmus*) at seven early developmental stages. Dissolved oxygen concentrations (mg/L) and survival curve for tra catfish at 2, 4, 6, 8, 9, 10, and 11 days post fertilization (dpf) **(A–G)**, during a low dissolved oxygen challenge. Dissolved oxygen level was reduced by replacing with oxygen stripping. Catfish were determined to be moribund when the opercular movement ceased.

A low-oxygen challenge experiment was conducted to test the survival ability of tra catfish larvae in anoxic conditions (0 ppm DO) as a function of age (Supplementary Table 1 and Figure 1; Backenstose, 2018). At 2 dpf, larval survival was 0% when the oxygen was lowered to 0 ppm (i.e., anoxia; Figure 2A). Initially, when the aeration was removed from the container, the larvae swam normally. After 15 min, when the DO level dropped below 2.1 ppm, the fish swam rapidly at the surface, showing a behavior indicative of oxygen stress. After 30 min, when the dissolved oxygen level in water dropped to 1.3 ppm, locomotion was dramatically dropped. After 45 min, when the dissolved oxygen level reached 0.7 ppm, the fish ceased to swim and sank to the bottom of the container; all of the fish were dead.

At 4 dpf (Figure 2B), the oxygen concentration fell rapidly from 5 to 1.5 ppm in 15 min after removing the aeration, and fish swam slowly. After 30 min, the dissolved oxygen level dropped to 1.1 ppm, and the fish swam rapidly at the surface. At 45 min, the dissolved oxygen levels decreased to 0 ppm, and 50% of the fish swam to the bottom of the tank, while no fish died at this point. After 55 min, with the dissolved oxygen concentration remaining at 0 ppm, all larvae were dead. We observed similar fish behavior for challenges at 6 and 8 dpf, and the ability to survive in the air increased gradually, but no fish survived the entire 75-min experiment (Figures 2C,D). However, at 9, 10, and 11 dpf, a large proportion of fish remained alive after 120 min, with a 60, 80, and 100% survival rate, respectively, (Figures 2E–G).

In conclusion, at a temperature of 27°C, within the first 6 dpf, tra catfish larvae displayed stress behavior when the DO dropped below 2 ppm for 15 min. Their survival was 0% when oxygen was reduced to and maintained at 0 ppm for 25 min. However, 4- and 6-dpf larvae already possessed some ability to survive hypoxic oxygen (0.7–1.1 ppm) and sometimes exhibited air-gulping behavior, which indicated that some of the tra catfish larvae had reached a point and developed the ability to obtain gas exchange activity through air-breathing. At 8 dpf, tra catfish larvae had 100% survival rate at DO = 0.6 ppm and could survive for a while at 0 ppm DO. The survival rate of tra catfish larvae was 60, 80, and 100% in 9, 10, and 11 dpf, respectively, when the challenge concluded after 120 min, indicating that at 9 and 10 dpf, the air-breathing ability was well developed. At 11 dpf, the swim bladder in tra catfish was fully developed, and tra catfish possessed complete air-breathing capability.

### Histological Analysis

**FIGURE 3:**
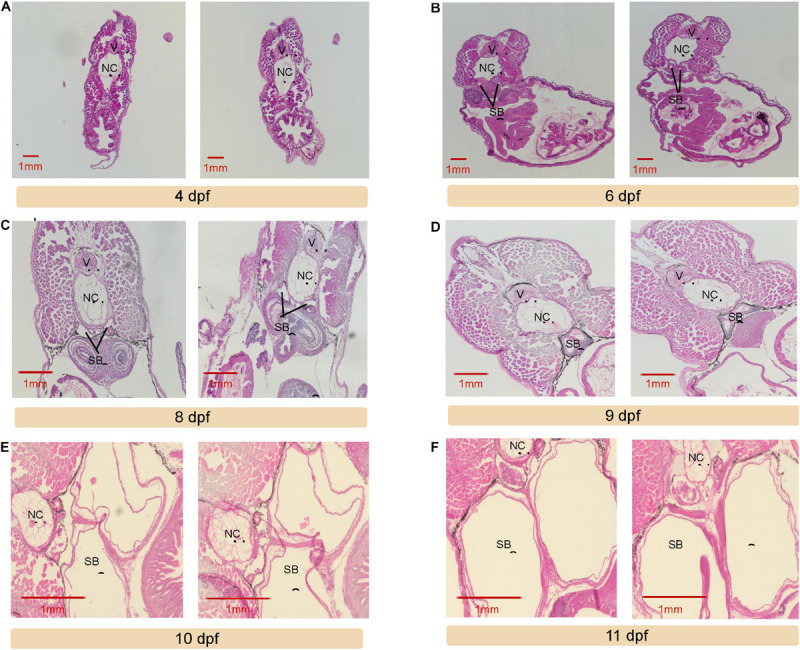
Transverse section for seven early development stages in *Pangasianodon hypophthalmus*. The transverse section is 7 μm thick, from tra catfish (*P. hypophthalmus*) 4 days post fertilization (dpf) to 11 **(A–F)**, including the vertebrae (V), notochord (NC), and swim bladder (SB). The vertebral column (V) was located just dorsally to the NC and was surrounded by musculature. The developing SB was distinctly bilobed.

At 4 dpf, the vertebrae column, the notochord, and vertebral column were observed at the central location of the front ventral surrounded by skeletal muscle. A small cavity formed within the yolk sac that was surrounded with simple gastrointestinal structures (Figure 3).

At 6 dpf, a moderate increase in the dimensions was found in the developing internal organs. The yolk sac was absorbed entirely at this point, and the larvae possessed the ability to swim freely and could regulate their orientation underwater. The newly observed swim bladder was situated retroperitoneally, located ventral to the notochord and dorsal to the peritoneal cavity, encompassing a significant portion of the body cavity. At this point, the tra larvae already had improved survival ability at 0.7 ppm DO, which is a hypoxic condition.

The swim bladder and other internal organs were further developed at 8 days post fertilization. The swim bladder increased slightly in size, and two distinct lobes were present, divided by a longitudinal, central septum. The ventral surface of the gas bladder was located outside of the visceral cavity and was attached to the parietal peritoneum. During this stage, survival in hypoxic conditions was slightly increased, as they could live in 0.6 ppm DO and could survive for a short period of time under anoxia.

At 9 dpf, the inner layer of the swim bladder consisted of a cuboidal epithelium, and the smooth outer layer consisted of an elastic collagenic fibrosa. The tra larvae possessed a long-term survival rate of 60% at 0 mg/L DO. At 10 dpf, there were distinct changes to swim bladder morphology. The musculature was slightly thicker. The swim bladder had expanded significantly; the inner layers pushed to the outer perimeter of the organ. It was extremely dilated and presumably engorged with air. Survival rate improved to 80% at 0 ppm DO.

Tra catfish larvae at 11 dpf were similar in microstructure to fish at 10 dpf. However, the swim bladder expanded significantly in size and reached the outer limits of the body cavity. The bilobed structure was demarcated by a protuberant central obstruct. Larvae at this stage had 100% survival rate during anoxia challenge. In conclusion, from 6 dpf, the tra catfish swim bladder served as an ABO, and aerial breathing ability was fully functional 5 days later, at 11 dpf.

### Comparison of Genomics of Tra Catfish and Channel Catfish

Tra catfish and channel catfish are both Siluriformes, but the air-breathing ability varies significantly between these two species. The tra catfish is a facultative air-breather and utilizes the swim bladder as its ABO in a low-oxygen environment, while channel catfish do not possess an ABO and thus cannot breathe in the air.

We sought to determine which genes would be involved in the development of air-breathing in tra catfish. Thus, we first compared the gene contents between tra catfish and channel catfish and discovered that a total of 76 genes present in tra catfish were absent from channel catfish (Supplementary Table 2). Many traits differ between tra catfish and channel catfish, not only the ability to breathe in the air. However, many genes should be involved in the formation of the swim bladder in tra catfish and the air-breathing ability. In this regard, genes that contribute to the swim bladder development and aerial breathing ability should be differentially expressed during tra catfish development. Thus, genes that are (1) present in tra catfish but absent from channel catfish and (2) differentially expressed during tra catfish development could be key genes involved in the morphogenesis of the swim bladder and differences in aerial breathing ability.

### Sequencing and Global Analysis of Tra Catfish Transcriptome

A total of 1,303 million raw reads were generated for tra catfish through RNA-seq analysis. As shown in Supplementary Table 3, after removing low-quality reads and trimmed reads that are less than 36 bases, approximately 587 million high-quality reads were retained from tra catfish. All clean reads were aligned to the tra catfish reference genome GENO_Phyp_1.0 (GenBank assembly accession GCA_009078355.1) using STAR software (v 2.7.0; Supplementary Figure 1).

**FIGURE 4:**
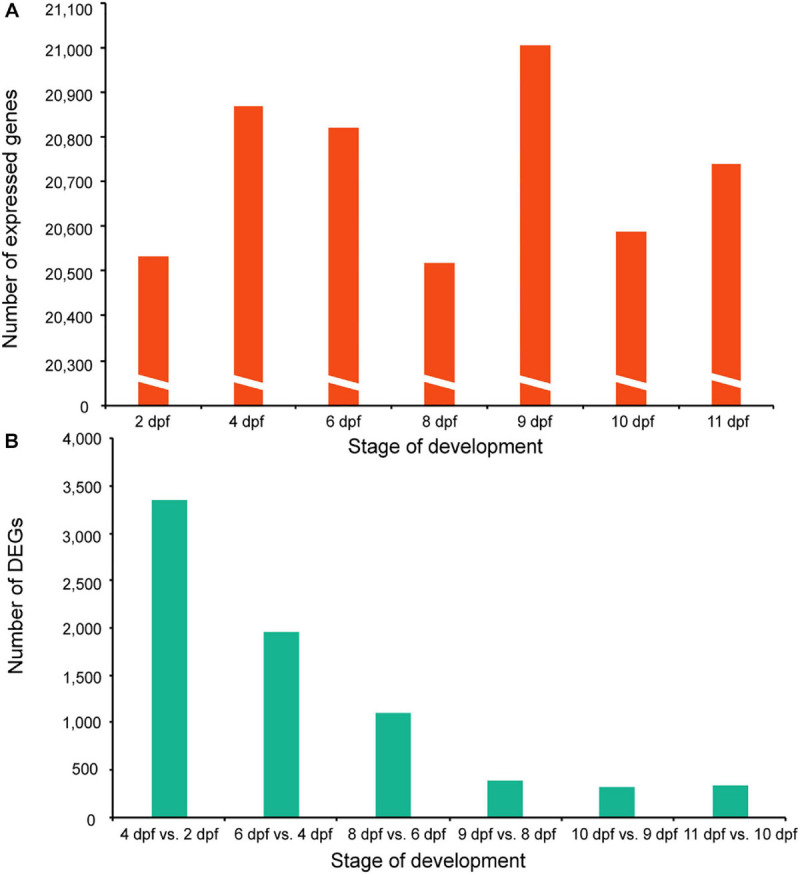
Summary of gene expression during early development in tra catfish (*Pangasianodon hypophthalmus*). **(A)** The number of expressed genes at each development stage averaged for two replicates. **(B)** The number of DEGs (differentially expressed genes) for comparison of each stage with the previous stage.

**FIGURE 5:**
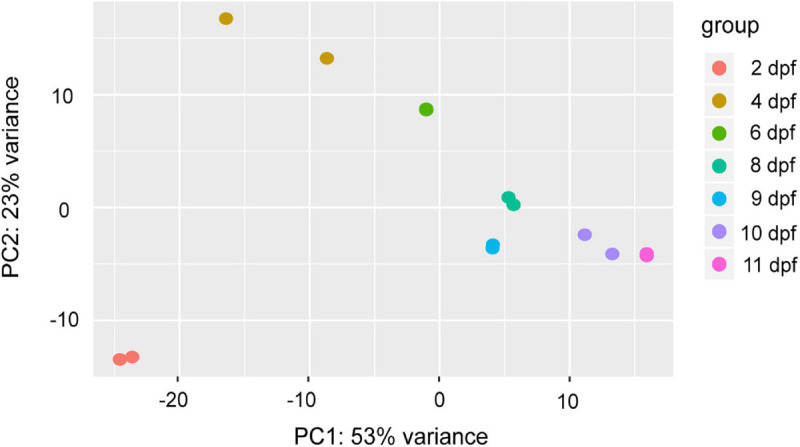
Principal component analysis (PCA) of tra catfish (*Pangasianodon hypophthalmus*) transcriptome (two replicates with some giving the exact same value).

Reads were assigned to transcripts by their overlaps with tra catfish reference gene models. The fragments per kilobase of exon model per million reads mapped (FPKM) method was performed and an FPKM cutoff for the expressed genes was set at ≥0.1. As shown in Figure 4A, a total of 21,448 transcripts were detected in the RNA-seq dataset, the highest number of expressed genes (21,004) was detected at 9 dpf, and the lowest number of expressed genes (20,516) was at 8 dpf. A total of 19,728 genes were discovered to be expressed in all samples (Supplementary Table 4). Principal component analysis (PCA) was used to identify the outliers in the tra catfish transcriptomes during different development time points (Figure 5). The clustering results were in agreement with the developmental stage groups. In Figure 5, the expression profiles of different time points were divided into seven clusters from left to right, with biological replicates within the group clustered together. We could observe considerable expression variability across different developmental points, accounting for 53% of expression variation.

### Differentially Expressed Genes During Developmental Stages of Tra Catfish

RNA sequencing reads were aligned with the tra catfish genome and gene read counts were calculated using HTSeq. DESeq2 software was used to identify the significantly and DEGs with a comparison of each stage to the previous stage. In tra catfish, the number of DEGs ranged from 3,360 (2,324 upregulated, 1,036 downregulated) between 4 and 2 dpf to 338 (213 upregulated, 125 downregulated) between 11 and 10 dpf (Supplementary Table 5). In general, the number of identified DEGs decreased during the development stages in tra catfish (Figure 4B); 1,956 (861 upregulated, 1,095 downregulated), 1,108 (661 upregulated, 447 downregulated), 380 (209 upregulated, 171 downregulated), and 324 (209 upregulated, 115 downregulated) DEGs were detected between 6 and 4 dpf, 8 and 6 dpf, 9 and 8 dpf, and 10 and 9 dpf, respectively. Taken together, 5,419 DEGs were detected at different developmental stages. Notably, the greatest number of DEGs was identified between the first two time points (4 and 2 dpf), which is not surprising since the transition from fertilized egg to hatchling is dramatic, and a particularly dynamic rearrangement of biological processes would be expected to occur between these stages. We then compared the identified 76 tra catfish-specific genes with the 5,419 DEGs during tra catfish development. Finally, 24 DEGs present in tra catfish but absent from channel catfish were illustrated (Figure 1 and Supplementary Table 6). We conclude that this collection of 24 genes may be closely related with the formation of air-breathing ability in tra catfish.

### Gene Ontology Enrichment Analysis of Differentially Expressed Genes at Different Stages

**FIGURE 6:**
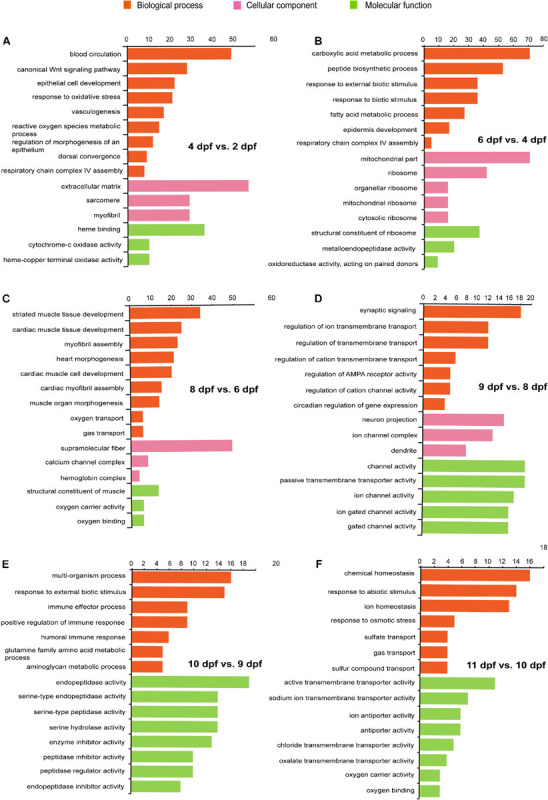
Gene ontology enrichment of DEGs at different development stages in tra catfish (*Pangasianodon hypophthalmus*). **(A)** 4 vs. 2 dpf. **(B)** 6 vs. 4 dpf. **(C)** 8 vs. 6 dpf. **(D)** 9 vs. 8 dpf. **(E)** 10 vs. 9 dpf. **(F)** 11 vs. 10 dpf. The vertical axis indicates the number of DEGs between two adjacent sampling datasets, and the horizontal axis represents the GO terms significantly enriched in the DEGs.

To determine the GO enrichment with related functions, GO enrichment analysis of DEGs was performed for each developmental stage (Figure 6 and Supplementary Table 5).

At 4 dpf compared with 2 dpf, the significantly enriched categories for the DEGs were mainly related to blood vessel formation, respiratory chain complex assembly, reactive oxygen species (ROS), and Wnt signaling pathway. In short, all of these processes were associated with vasculogenesis (GO:0001570). Day 4 was also associated with respiratory chain complex IV assembly (GO:0008535) and regulation of ROS metabolic process (GO:2000377). Several mitochondrial activities and respiratory chain functions were enriched at 6 dpf compared with 4 dpf, including respiratory chain complex IV assembly (GO:0008535), mitochondrial inner membrane (GO:0005743), mitochondrial ribosome (GO:0005761), and mitochondrial protein complex (GO:0098798).

At 8 dpf compared with 6 dpf, enriched categories for the DEGs included genes related to the morphogenesis of heart and muscle. However, they were notably involved with processes that would be expected to be associated with improved oxygen transport. These included heart morphogenesis (GO:0003007), muscle organ morphogenesis (GO:0048644), oxygen transport (GO:0015671), oxygen carrier activity (GO:0005344), oxygen binding (GO:0019825), calcium ion transmembrane transporter activity (GO:0015085), hemoglobin complex (GO:0005833), and cardiac myofibril assembly (GO:0055003). At 9 dpf compared with 8 dpf, DEGs were mainly enriched in transmembrane transport activity, dendrite, and some channel activity. The activities include regulation of ion transmembrane transport (GO:0034765), regulation of cation transmembrane transport (GO:1904062), transmembrane transporter complex (GO:1902495), dendrite membrane (GO:0032590), dendritic tree (GO:0097447), calcium channel complex (GO:0034704), potassium channel complex (GO:0034705), and ion gated channel activity (GO:0022839).

At the 10-dpf compared with the 9-dpf stage, the DEGs were mainly enriched in the metabolic process and endopeptidase activity, including glutamine family amino acid metabolic process (GO:0009064), aminoglycan metabolic process (GO:0006022), serine-type endopeptidase activity (GO:0004252), peptidase regulator activity (GO:0061134), and carboxylic ester hydrolase activity (GO:0052689). Additionally, DEGs from 11 dpf compared with 10 dpf were enriched in oxygen and ion transport and ATP activity, including oxygen carrier activity (GO:0005344), oxygen binding (GO:0019825), bicarbonate transport (GO:0015701), sulfur compound transport (GO:0072348), sodium ion transmembrane transporter activity (GO:0015081), anion:anion antiporter activity (GO:0015301), chloride transmembrane transporter activity (GO:0015108), ATPase activator activity (GO:0001671), and sodium:potassium-exchanging ATPase activity (GO:0005391).

### Gene Expression Profiling of Tra Catfish-Specific Genes

**FIGURE 7:**
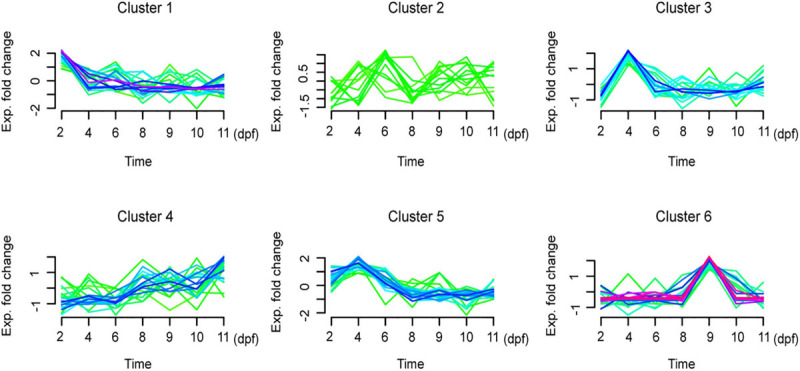
Time series expression profiles for tra catfish (*Pangasianodon hypophthalmus*) specific genes. These tra catfish-specific genes are grouped into six clusters. dpf: days post fertilization.

To further narrow down the list of the candidate key genes that may have a key role in the development of the swim bladder and on the function of aerial breathing, the expression model of the 76 tra catfish-specific genes were drawn according to the total FPKM value at each development stage, and a clear variation was observed (Figure 7). The 76 tra catfish-specific genes were categorized into six different clusters (Supplementary Table 7). A total of 48 genes in clusters 1, 3, and 5 showed a peak of expression at 2 or 4 days post fertilization and then decreased. Cluster 6 (eight genes) showed a peak of expression values at 9 dpf, which is the date just before the swim bladder increased enormously in size. Also, there were 12 genes in cluster 4, showing an increasing expression profile, and 8 genes in cluster 2 for which the expression profile remained unchanged during the development of tra catfish.

Furthermore, from the previous histology experiment, we investigated that the swim bladder can be observed in tra catfish larvae from 6 dpf, and the 0-ppm oxygen challenge experiment revealed that tra catfish larvae already possessed partial aerial breathing ability even before that. However, both the hypoxic challenge experiment and histology experiment illustrated that the fish did not possess full air-breathing ability until 11 dpf. Thus, for gene expression profiles, the candidate key genes, which contributed to swim bladder development and air-breathing function would be expected to be turned on before 6 dpf (maybe at 4 dpf or as early as 2 dpf) or at least to have increasing expression with the formation of air-breathing function over time. In addition, the genes with peak production at 9 dpf may contribute to the rapid expansion of the structure of ABO and consequently the air-breathing ability at 11 dpf. In this regard, we presumed that the genes in clusters 1, 3, 4, 5, and 6 have the greatest likelihood to play an important role in swim bladder development and air-breathing. Based on these criteria, we already identified 24 genes to be tra catfish-specific genes as well as differentially expressed during tra catfish development (Supplementary Table 6). These genes were summarized in groups based on their expression profiles (Table 1). Excluding genes with unknown function, we are left with 14 genes in clusters 1, 3, 4, 5, and 6 as the best candidates of air-breathing function-related genes in tra catfish: PET117 Cytochrome C Oxidase Chaperone (*PET117*); spidroin-2-like isoform X1 (*SPIDROIN*); gastrin-releasing peptide-like isoform X2 (*GRP*); chromo domain-containing protein cec-1-like isoform X2 (*CX3CL1*); lymphocyte antigen 6D (*LY6D*); dual-specificity protein kinase shkD-like isoform X2 (*SHKD*); non-compact myelin-associated protein (*NCMAP*); calcium-activated chloride channel regulator 3A-1-like (*CLCA3A1*); calcium-activated chloride channel regulator 1-like (*CLCA1*); histidine-rich glycoprotein-like (*HRG*); cytochrome c oxidase subunit 7C, mitochondrial (*COX7C*); zinc finger protein 862-like (*ZNF862*); L antigen family member 3 (*LAGE3*); and protein FAM216A isoform X1 (*FAM216A*).

**TABLE 1:** Twenty-four key candidate genes.

**Cluster**	**Gene name**	**Gene ID**	**Gene description**
Cluster 1	PET117	113547415	PET117 Cytochrome C Oxidase Chaperone
	SPIDROIN	113547475	Spidroin-2-like isoform X1
Cluster 2	CUNH3ORF85	113531560	Uncharacterized C3orf85 homolog
	VXN	113541042	Vexin-like
	LOC113542136	113542136	Uncharacterized protein LOC113542136
Cluster 3	GRP	113535796	Gastrin-releasing peptide-like isoform X2
	CL3CL1	113533685	Chromo domain-containing protein cec-1-like isoform X2
	LY6D	113539350	Lymphocyte antigen 6D
	SHKD	113533778	Dual-specificity protein kinase shkD-like isoform X2
	NCMAP	113545006	Non-compact myelin-associated protein
	LOC113534207	113534207	Uncharacterized protein LOC113534207
Cluster 4	CLCA3A1	113544546	Calcium-activated chloride channel regulator 3A-1-like
	CLCA1	113544571	Calcium-activated chloride channel regulator 1-like
	LOC113541665	113541665	Uncharacterized protein LOC113541665
	LOC113542106	113542106	Uncharacterized protein LOC113542106 isoform X1
	LOC113546726	113546726	Uncharacterized protein LOC113546726
Cluster 5	HRG	113547643	Histidine-rich glycoprotein
	COX7C	113536891	Cytochrome c oxidase subunit 7C, mitochondrial
	ZNF862	113539654	Zinc finger protein 862-like
	LAGE3	113544929	L antigen family member 3
	*FAM216A*	113535709	Protein FAM216A isoform X1
	LOC113538378	113538378	Uncharacterized protein LOC113538378
Cluster 6	LOC113524458	113524458	Uncharacterized protein LOC113524458
	LOC113543977	113543977	Uncharacterized protein LOC113543977

*The key genes were grouped into six clusters based on their expression profiles during development in tra catfish (*Pangasianodon hypophthalmus*).*

## Discussion

Life evolved on Earth that experienced “aperiodic” hypoxia (Föllmi, 2012), but today, aerobic respiration is critical for efficient energy metabolism, a precondition for the beginning of complex creatures (Catling et al., 2005). Various respiratory systems have evolved to obtain oxygen from the environment. For water-breathing fish, gills are used as the chief gas exchangers, while it is thought that the reduction of dissolved aquatic oxygen promoted the migration of life from water to land and compelled the evolution of water-breathers to air-breathers (Hsia et al., 2013). Amphibians, some turtles, and mammals have been theorized to have undergone key evolutionary processes associated with the move to land (Owen, 1855; Tokita et al., 2013; Radinsky, 2015). In the intermediary step of aquatic to terrestrial breathing, the ABOs have evolved into many different forms in different fish, including modified gills, skins, trachea, intestine, and swim bladder. The swim bladder has long been postulated as a homolog of the lung in terrestrial vertebrates (Owen, 1855). The swim bladder of zebrafish arose from branches of the foregut endoderm, close to the liver and pancreas, which compares well with mammalian lung (Spooner and Wessells, 1970; Field et al., 2003). The Wnt signaling pathways were found to play a critical role in the development of the zebrafish swim bladder as well as the vertebrates’ lung (Yin et al., 2010, 2011; Yang et al., 2018). Moreover, Zheng et al. (2011) reported a strong resemblance between the zebrafish swim bladder and mammalian lungs by comparison of their transcriptomes. These studies suggest that mammalian lungs may have originated from the teleost swim bladder, and genes that contributed to the function of the lungs may also be critical for the formation of air-breathing ability in fish.

In this study, low to zero oxygen challenge experiments and histology were combined to reveal the development of air-breathing capabilities in concert with the formation of the swim bladder in tra catfish. We sequenced and analyzed the transcriptome from seven key developmental stages in tra catfish, providing a comprehensive understanding of this species and their unique ability in air-breathing. A total of 5,419 DEGs were identified during the early development of tra catfish. GO enrichment analysis revealed that these genes were mainly concerned with blood vessel formation, respiratory chain complex, transportation and binding of oxygen, transmembrane activities, and in general, processes associated with air-breathing. We then compared the genomic contents of channel catfish and tra catfish, which belong to the same order (Siluriformes). 76 unique genes were identified that were active in tra catfish, but were absent from channel catfish. Our study suggests that they might play key roles in the development of the swim bladder and air-breathing functions in tra. Gene expression analysis was also performed for tra catfish-specific genes, and 14 genes were selected and believed to be implicated in the air-breathing ability in tra catfish. A similar analysis was performed using Illumina RNA sequencing for the six developmental stages (4, 8, 12, 20, and 40 days post hatch and 1 year old) of the posterior intestine, the ABO of dojo loach (*M. anguillicaudatus*; Luo et al., 2016). According to the differential expression analysis among different developmental stages and gene expression analysis, 25 key genes were detected to be potential target genes involved in the formation of intestinal air-breathing function in *M. anguillicaudatus*. These included *GSN*, *YES1*, *CISH*, *RHOA*, *PRKCE*, *RAF1*, *BAD*, *SOCS3*, *PIK3CA*, *AKT1*, *KDR*, *EGFR*, *TP53*, *JUN*, *SMAD4*, *PKT2*, *MAPK14*, *GRB2*, *VEGFA*, *MYC*, *TNF*, *DVL2*, *ROS1*, *ETV5*, and *FZD10* (Luo et al., 2016). However, none of these genes match the key ABO genes in our tra catfish study, which use the swim bladder instead of the intestine for air-breathing. Luo et al. (2016) also discussed that these genes were seldom characterized and attributed to air-breathing function in fish. These genes were mainly involved in development, angiogenesis, and cytoskeleton and thus considered to contribute to the intestinal aerial breathing function formed process during posterior intestine development in dojo loach (Luo et al., 2016). The lack of intersection among these 25 genes with the 14 genes we identified to be candidate key genes for the air-breathing function in tra catfish may emphasize that when dissimilar species use radically different organs for air-breathing, one can expect to see the use of very different genes to accomplish the task of air-breathing. Further studies on the two species using *in situ* hybridization may confirm these apparent differences. The putative functional roles and related pathways of the key genes are discussed below.

PET117 Cytochrome C Oxidase Chaperone (*PET117*) is a protein-coding gene. Diseases associated with *PET117* include mitochondrial complex IV deficiency and Charcot–Marie–Tooth disease, type 4K (Renkema et al., 2017). Human (*Homo sapiens*) *PET117* functions the same way in cytochrome c oxidase (*cox*) biogenesis as that in yeast (*Saccharomyces cerevisiae*), although further experimentation needs to provide conclusive evidence in mammalian systems (Szklarczyk et al., 2012). Vidoni et al. (2017) demonstrated that in the presence of *PET100*, *PET117* interacts with myofibrillogenesis regulator 1 (MR-1S) and with some COX subunits. Such interaction proved to assist COX biogenesis in higher eukaryotes (Vidoni et al., 2017).

*SPIDROIN-2*, one of the genes identified as upregulated in group 1, is also named Dragline silk fibroin 2, and is known as a silk protein. Sequencing analysis indicates that it belongs to the silk fibroin family, which is a highly repetitive protein characterized by regions of polyalanine and glycine-rich repeating units. In *Golden silk orbweaver* (*Nephila clavipes*), the spiders’ major ampullate silk possesses unique characteristics of strength and elasticity. Until recently, there has been little evidence of this gene reported in teleost fishes, but its specific function in aquatic species needs further investigation (Gaines and Marcotte, 2008; Correa-Garhwal et al., 2019).

Gastrin-releasing peptide is a bombesin-like peptide generated by pulmonary neuroendocrine cells (PNEC). Many studies report this gene to play a key role in PNEC growth and embryonic lung branching (Spurzem et al., 1997; Zheng et al., 2011). As reported by Spindel et al. (1987), *GRP* expression was elevated in human embryonic pulmonary systems during the canalicular phase of lung development (16 to 30 weeks post fertilization). Through RNA blot and *in situ* hybridization analyses, *GRP* expression was first detected in the fetal lung at 9–10 weeks, reaching a plateau from 16 to approximately 30 weeks, which was 25-fold higher than in mature lungs, and then declined to an adult level at 34 weeks postpartum. By contrast, *GRP* peptide levels remained elevated until several months after birth. The transient high-level expression of *GRP* during nearly a 12-week phase of embryonic lung development suggested that the secretion of *GRP* or its COOH-terminal peptides from PNECs might be closely associated with normal lung development in humans (Spindel et al., 1987). *In situ* hybridization in human embryos during early pregnancy indicated that *GRP* had the highest expression first in the proximal lung and that as the lung continued to develop, the intensity of expression increased in the distal lung. This suggests that that *GRP* was activated as part of the normal progression from a proximal to distal development, consistent with the differentiation and development of respiratory bronchioles. These related observations suggest that *GRP* plays an important role in human fetal lung development. In addition, the *GRP* gene is necessary for inducing the formation of primitive air saccules along respiratory bronchioles as well as the continuing extension of airway epithelium (Spindel et al., 1987). In 1984, Uddman reported that *GRP* peptide might also regulate local blood flow, glandular secretion, and the activity of smooth muscle (Uddman et al., 1984). Martínez and coworkers found that the addition of *GRP* in human cells could increase endothelial cell migration and cord formation as well as induce angiogenesis *in vitro* (Martínez et al., 2005).

Gastrin-releasing peptide was reported to have similar distribution and function in mice (*Mus musculus*) as humans. There is evidence that *GRP* is associated with fetal mouse lung branching during morphogenesis (Aguayo et al., 1994). In mice, *GRP* and *GRP* receptor genes are expressed in the embryonic lung as early as embryonic day 12, when the lung begins to branch (Aguayo et al., 1994). *In situ* hybridization in mice revealed that the *GRP* receptor reaches its highest level of expression in mesenchymal cells at cleft regions of branching airways and blood vessels (Aguayo et al., 1994; King et al., 1995).

Fractalkine-like isoform X1 (*CX3CL1*) is another gene we identified in our developmental time series analysis, which is potentially important in the development of swim bladder and air-breathing function. In human pulmonary circulation, high blood flow and low pressure were preserved by distal arterioles with limited smooth muscles (Zhang et al., 2012). Human lung increases the air exchange area and capillary length to compensate pulmonary alveolar. Also, the lung microvascular endothelium produces excessive *CX3CL1* in response to hypoxia, which could stimulate phenotypic switching, proliferation, and muscle expansion in SMC (Zhang et al., 2012). Though probably related to hypoxia tolerance, little is known about this gene in fish.

The lymphocyte antigen 6 (*LY6*) gene family belongs to the superfamily of lymphocyte antigen-6 (*LY6*)/urokinase-type plasminogen activator receptor (uPAR) proteins (Consortium, 2015). This superfamily is characterized by a LU domain (60–80 amino acids), which is composed of 6–10 cysteines arranged in a specific spacing pattern that allows distinct disulfide bridges that create the ancient three-fingered (3F) structural motif (Consortium, 2015). Lymphocyte antigen 6D (*LY6D* or *Ly-6D* or *E48*) in humans is involved in cell adhesion, lymphocyte differentiation, and response to stilbenoid (Bateman et al., 2004). It can be used as a molecular marker to distinguish between B- and T-cell lymphocyte types at the earliest stage. This gene is expressed at the outer cell surface of translational epithelia and keratinocytes of stratified squamous epithelia, indicating tissue specificity (Consortium, 2015). A few human orthologs in the mouse of *LY6* include *LY6E*, *LY6K*, *Lynx1*, *Slurp1*, and *Gpihbp1*. Knockout of these genes results in embryonic lethality, infertility in male mice, increased visual cortex plasticity, palmoplantar keratoderma with metabolic and neuromuscular abnormal phenotypes, and hypertriglyceridemia phenotype. Most of the *LY6* homologs (i.e., *SCA1*, *LY6B*, *LY6C*, *LY6G*, *LY6I/LY6M*, and *LY6F*) in mice are expressed in immune cells, such as B cells, T cells, NK cells, monocytes, and dendritic cells, indicating involvement in the immune system (Upadhyay, 2019). Interestingly, *LY6K* from clinical data demonstrated that overexpression of *LY6K* leads to a few organ cancers, including breast cancer, esophageal squamous cancer, gingivobuccal cancers, bladder cancer, and lung cancer (Upadhyay, 2019). Therefore, we speculate that overexpression of *LY6* in fish may cause dysfunction of the swim bladder, gill, and other organs. However, in the current study, this transcript contig was too short to predict any biological structure.

Non-compact myelin-associated protein (*NCMAP*) has other alternative names, myelin protein 11 kDa, or *C1orf130*, or short for *MP11*, and plays a role in myelin formation (Safran et al., 2010). Diseases associated with *NCMAP* include Dieulafoy lesion and GO annotations related to this gene include structural constituents of the myelin sheath (Safran et al., 2010). It is a membrane protein that inhibits myelination when either over- or underexpressed. *MP11* expression is restricted to the placenta and peripheral nervous system, where it is expressed by Schwann cells and localized to paranodes and Schmidt–Lanterman incisures (*SLIs*) of non-compact myelin (Ryu et al., 2008).

Calcium-activated chloride channel regulator 1 and *CLCA4A1* both belong to part of a larger family of *CLCA* proteins that has conserved domain architectures, such as the *CLCA* domain, *VWA* domain, and transmembrane domain. *CLCA1* factor has widely been found in many epithelial cells, endothelial cells, and smooth muscle cells. Its main roles include chloride transport and mucin expression (Evans et al., 2004). The *VWA* name comes from the von Willebrand factor (*vWF*) type A domain. The von Willebrand factor is a large multimeric glycoprotein found in blood plasma, and mutation of *VWA* causes bleeding disorders (Ruggeri and Ware, 1993). *CLCA* family members have been reported in different species, including human (four genes from *hCLCA1* to *hCLCA4*), mouse and rat (eight homologs from *mCLCA1* to *mCLCA8*), and cow [*bCLCA1*, *bCLCA2* (Lu-ECAM-1), *bCLCA3*, and *bCLCA4*] (Liu and Shi, 2019). There are six ortholog genes documented in zebrafish: *CLCA1*, *CLCA5.1*, *CLCA5.2*, *CLCA1-201*, *CLCA1-203*, and *CLCA5.1-201* (Howe et al., 2012). In our current hypoxia study, two orthologs are found—*CLCA1* and *CLCA3*. Recent studies demonstrated that *CLCA1* forms non-covalent oligomers in colonic mucus and has Mucin 2-processing properties, playing an important role in regulating the structural arrangement of the mucus and thereby partly mediating mucus processing (Dayal et al., 2019; Nyström et al., 2019). Few reports are available on the functional analysis of *CLCA3*. However, *CLCA3* has conserved domains with *CLCA1* and shares 82% identity with each other using MatGATv program (data not shown; Companella et al., 2003). In addition, *CLCA3* has one specific domain, FN3, fibronectin type 3 domain, which is involved in cell adhesion, cell morphology, thrombosis, cell migration, and embryonic differentiation (Petersen et al., 1983).

Another candidate gene for air-breathing in tra was *HRG*. Li et al. (2018) reported that genes related to angiogenesis may be one of the adaptations for the ABO to retain the high efficiency of gas exchange and, thus, are one of the critical components for air-breathing fish to adapt to low-oxygen terrestrial conditions (Li et al., 2018). *HRG* is mainly present in plasma fluid and is thought to play various roles in the human blood, such as angiogenesis, vascularization, coagulation, and immunity (Leung et al., 1983). During angiogenesis, *HRG* binds to thrombospondin (TSP) and TSP-1, which is a powerful inhibitor of angiogenesis. *HRG* was reported to inhibit the antiangiogenic effect of *TSP-1* (Blank and Shoenfeld, 2008; Wakabayashi, 2013).

Cytochrome c oxidase is composed of 13 subunits, 3 encoded by mitochondrial (mt)DNA and 10 encoded by nuclear genes. *COX7C* is one of the last enzymes in the mitochondrial electron transport chain that drives oxidative phosphorylation. This respiratory chain catalyzes the reduction of oxygen to water. *COX7C*, *NRF1*, and *PGC1*α itself, in the putative *PGC1*α axis, showed no increase in mRNA in response to AMPK activation, while cold acclimation induced 4.1-fold increase in *COX* activity relative to warm-acclimated goldfish (Bremer et al., 2016). Indeed, low temperature induces mitochondrial biogenesis in many fish species (O’Brien, 2011). However, Duggan et al. (2011) showed that not all of the subunits of *COX* such as *COX4-1*, *COX5A1*, *COX6B1*, *COX6C*, and *COX7C* are cold-responsive genes in dace (*Chrosomus eos*), goldfish (*Carassius auratus*), and zebrafish (*Danio rerio*), suggesting coordination of *COX* gene expression in the remodeling of fish skeletal muscle.

Zinc Finger Protein 862 in humans functions in transcriptional regulation by binding metal ions and nucleic acid and, furthermore, has protein dimerization activity (Zhang et al., 2000). Few reports from fish could be found till now. This gene only has a partial cDNA sequence in our RNA-seq data.

L antigen family member 3 has a typical transmembrane domain and Pcc1 domain. The Pcc1 family is conserved and can be found in yeast (*S. cerevisiae*) as the EKC/KEOPS complex subunit Pcc1, and also in mammals, the EKC/KEOPS complex subunit *LAGE3*, and also as human cancer/testis antigen (CTAG) 1/2 (Bateman et al., 1999). Human (*H. sapiens*) lage3 is a homolog to ECK, which in both yeast and human is essential for tRNA modification activity (Kisseleva-Romanova et al., 2006; Costessi et al., 2012).

Family with sequence similarity 216 member A (*FAM216B*) is predicted to have a domain *FAM216B*. Its family members are approximately 150–270 amino acids in length. In humans, the *FAM216B* protein is encoded by C13orf30. In *P. hypophthalmus*, *FAM216A* has 180 amino acids in length and shows 43.75% identity to zebrafish (data not shown) and 39% to humans (NCBI blast). The function of this gene is not well identified.

vexin-like *(VXN)* gene belongs to cluster 2, which was a cluster differentially expressed and assumed to be involved in air-breathing. However, *VXN* gene expression does not change over time and likely does not appear to be associated with air-breathing ability.

All 14 characterized genes, excluding *VXN*, are very likely closely related to the formation of air-breathing ability in tra catfish. Of course, *HRG*, *GRP*, and *CX3CL1* are the most important candidate genes, as they were reported to be critical for the formation and function of human lung and angiogenesis.

## Conclusion

Tra catfish are aquatic but can use their swim bladders to breathe air, while channel catfish cannot perform air-breathing directly. As such, these two species provide excellent contrasting models to study the transition from aquatic to terrestrial living and the genes that are critical for the development of swim bladder, as well as the function of air-breathing in tra catfish. Through comparative genetic analysis between tra catfish and channel catfish, 76 genes were initially and uniquely identified to be in tra catfish but were absent from channel catfish. Hypoxia challenge and histology experiments revealed the critical time points for the air-breathing ability and swim bladder development in tra catfish. Further analysis was performed to narrow down the list of key candidate genes for air-breathing. Fourteen genes that play important roles in the formation of air-breathing ability in tra catfish were ultimately selected. *HRG*, *GRP*, and *CX3CL1* were confirmed to be critical for human lung growth, maintenance of lung function, and angiogenesis and for improving respiratory efficiency, suggesting that these genes may play important roles for the functioning of air-breathing ability in tra catfish. Further study should include the application of *in situ* hybridization, overexpression, and knockout of selected genes to verify their cellular and molecular mechanisms underlying air-breathing functions in vertebrates and tra catfish.

## Data Availability Statement

The data presented in the study are deposited in the NCBI repository (https://www.ncbi.nlm.nih.gov/geo/), accession number GSE154904.

## Ethics Statement

The animal study was reviewed and approved by Auburn University and Can Tho University Institutional Animal Care and Use Committee.

## Author Contributions

XM: investigation, methodology, supervision, software, formal analysis, visualization, and writing—original draft. MS: investigation. BS: writing—review and editing and supervision. AW: methodology. MB: methodology. VA: investigation. RS: visualization. MN: investigation. NB: conceptualization. AM: methodology and writing—review and editing. T-YD: investigation. XW: software, writing—review and editing, visualization, supervision, and funding acquisition. RD: conceptualization, writing—review and editing, supervision, project administration, and funding acquisition. All authors contributed to the article and approved the submitted version.

## Conflict of Interest

The authors declare that the research was conducted in the absence of any commercial or financial relationships that could be construed as a potential conflict of interest.

## Supplementary Material

Supplementary Figure 1 Overview of RNA-Seq mapping in tra catfish (*Pangasianodon hypophthalmus*).

Supplementary Table 1 Results of hypoxia challenge under direct exposure to air for *Pangasianodon hypophthalmus* at 7 early developmental stages.

Supplementary Table 2 Genes specific in tra catfish (*Pangasianodon hypophthalmus*).

Supplementary Table 3 Summary statistics of the transcriptome sequencing in tra catfish (*Pangasianodon hypophthalmus*) across early development stages [PanN1-N2; sample and replicate of tra catfish days post-fertilization (dpf)- replicate number].

Supplementary Table 4 Normalized gene read counts in tra catfish (*Pangasianodon hypophthalmus*) across 7 developmental stages.

Supplementary Table 5 Differentially expressed genes (DEGs) in *Pangasianodon hypophthalmus* across 7 early developmental stages.

Supplementary Table 6 Genes specific to tra catfish (*Pangasianodon hypophthalmus*) that were differentially expressed during tra catfish development.

Supplementary Table 7 Clustering of gene expression profiles for tra catfish (*Pangasianodon hypophthalmus*) specific genes. These tra catfish specific genes were grouped into 6 clusters.
