# Supplementary figures and images for: Comparative Transcriptome Analysis During the Seven Developmental Stages of Channel Catfish (Ictalurus punctatus) and Tra Catfish (Pangasianodon hypophthalmus) Provides Novel Insights for Terrestrial Adaptation
Source: Front Genet. 2021 Jan 21;11:608325. doi: 10.3389/fgene.2020.608325 (PMC7859520; doi:10.3389/fgene.2020.608325)

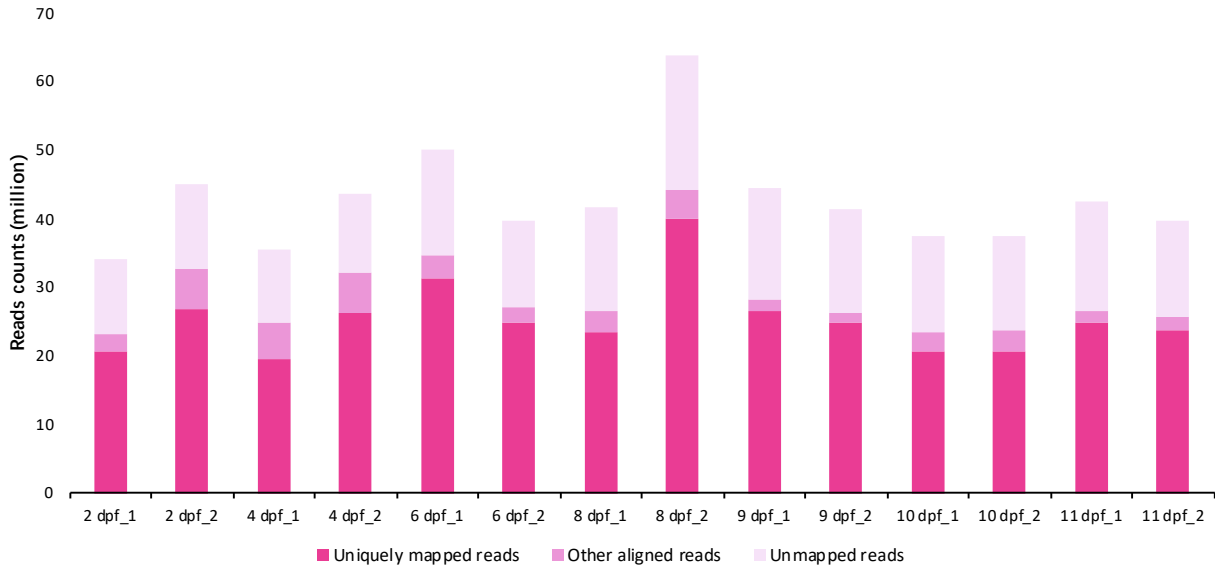

Supplement: Supplementary Figure 1 — Overview of RNA-Seq mapping in tra catfish (Pangasianodon hypophthalmus). [file Image_1.pdf]
